# Supplementary material for: Asynchronous magnetic resonance elastography: Shear wave speed reconstruction using noise correlation of incoherent waves
Source: Magn Reson Med. 2022 Oct 27;89(3):990–1001. doi: 10.1002/mrm.29502 (PMC9792433; doi:10.1002/mrm.29502)
Supplement: Supplementary file 1 — DATA S1 MATLAB code used to generate the numerical simulations of Figure 1 [file MRM-89-990-s002.zip › k-Wave/helpfiles/example_pr_2D_tr_iterative.html]

Iterative Image Improvement Using Time Reversal Example (k-Wave)


# Iterative Image Improvement Using Time Reversal Example

This example demonstrates how photoacoustic image reconstruction may be improved iteratively using time reversal and a positivity condition. This example builds on the 2D Time Reversal Reconstruction For A Line Sensor Example.

- Open the file in the MATLAB Editor
- Run the file in MATLAB

## Contents

- Simulating the sensor data
- Image reconstruction using time reversal
- Iterating to improve the image reconstruction

## Simulating the sensor data

The example begins by defining an initial pressure distribution using a pre-defined image of the word 'k-Wave'. The acoustic pressure time series measured at each of the sensor positions in an L-shaped sensor array are then simulated using `kspaceFirstOrder2D`. The reason for using an L-shaped detector is so there will be a 'visible region' (the convex hull) in which the iterations below will converge to the true solution.

```
% load an image for the initial pressure distribution
p0_image = loadImage('EXAMPLE_k-Wave.png');

% make it binary
p0_image = double(p0_image > 0);

% smooth the initial pressure distribution
p0 = smooth(p0_image, true);

% assign to the source structure
source.p0 = p0;
```

## Image reconstruction using time reversal

The simulated data is then used in a time reversal image reconstruction. One way to setup time reversal is described in 2D Time Reversal Reconstruction For A Line Sensor Example. Here, a different way to setup time reversal is described, in which the time series are explicitly reversed in time and the `source.p_mode = 'dirichlet'` option is used to enforce the values at the sensor points.

```
% use the sensor points as sources in time reversal
source.p_mask = sensor.mask;

% time reverse and assign the data
source.p = fliplr(sensor_data);	

% enforce, rather than add, the time-reversed pressure values
source.p_mode = 'dirichlet';    

% set the simulation to record the final image (at t = 0)
sensor.record = {'p_final'};

% run the time reversal reconstruction
p0_estimate = kspaceFirstOrder2D(kgrid, medium, source, sensor, input_args{:});

% apply a positivity condition
p0_estimate.p_final = p0_estimate.p_final .* (p0_estimate.p_final > 0);
```

Note that a positivity condition is applied to the reconstructed image because, from physical considerations, the initial acoustic pressure distribution must be zero or greater. The initial pressure distribution and image resulting from this first reconstruction are shown below.


## Iterating to improve the image reconstruction

Beginning with the image obtained using time reversal, an iterative procedure is then used to improve it. First, the image from the first step is used as a photoacoustic source in a forward model. The resulting time series are subtracted from the original measured time series to give a residual.

```
% set the initial pressure to be the latest estimate of p0
source.p0 = p0_estimate.p_final;

% set the simulation to record the time series
sensor = rmfield(sensor, 'record');

% calculate the time series using the latest estimate of p0
sensor_data2 = kspaceFirstOrder2D(kgrid, medium, source, sensor, input_args{:});

% calculate the error in the estimated time series
data_difference = sensor_data - sensor_data2;
```

An image is then formed from this residual data using time reversal reconstruction in the usual way. The resulting image is then added to the original image to give an improved estimate.

```
% assign the data_difference as a time-reversal source
source.p_mask = sensor.mask;
source.p = fliplr(data_difference);
source = rmfield(source,'p0');
source.p_mode = 'dirichlet';   

% set the simulation to record the final image (at t = 0)
sensor.record = {'p_final'};

% run the time reversal reconstruction
p0_update = kspaceFirstOrder2D(kgrid, medium, source, sensor, input_args{:});

% add the update to the latest image
p0_estimate.p_final = p0_estimate.p_final + p0_update.p_final;

% apply a positivity condition
p0_estimate.p_final = p0_estimate.p_final .* (p0_estimate.p_final > 0);
```

This simulation-reconstruction loop can then be repeated until there is no further noticeable improvement in the image. The image after three iterations is shown below.

Notice that within the region in which the visibility condition is satisfied (above the dashed line) the amplitude has recovered the correct value and the artefacts have reduced, but outside this region the amplitude remains low and there are still artefacts. This could be improved by recording data in more positions around the object, thereby increasing the visible region, or making other a priori assumptions about the image. In the latter case, an alternative approach to image reconstruction may be required. See Iterative Image Reconstruction Using the Adjoint Example.
